# Supplementary figures and images for: Bulked segregant analysis RNA-seq (BSR-Seq) validated a stem resistance locus in Aegilops umbellulata, a wild relative of wheat
Source: PLoS One. 2019 Sep 20;14(9):e0215492. doi: 10.1371/journal.pone.0215492 (PMC6754143; doi:10.1371/journal.pone.0215492)

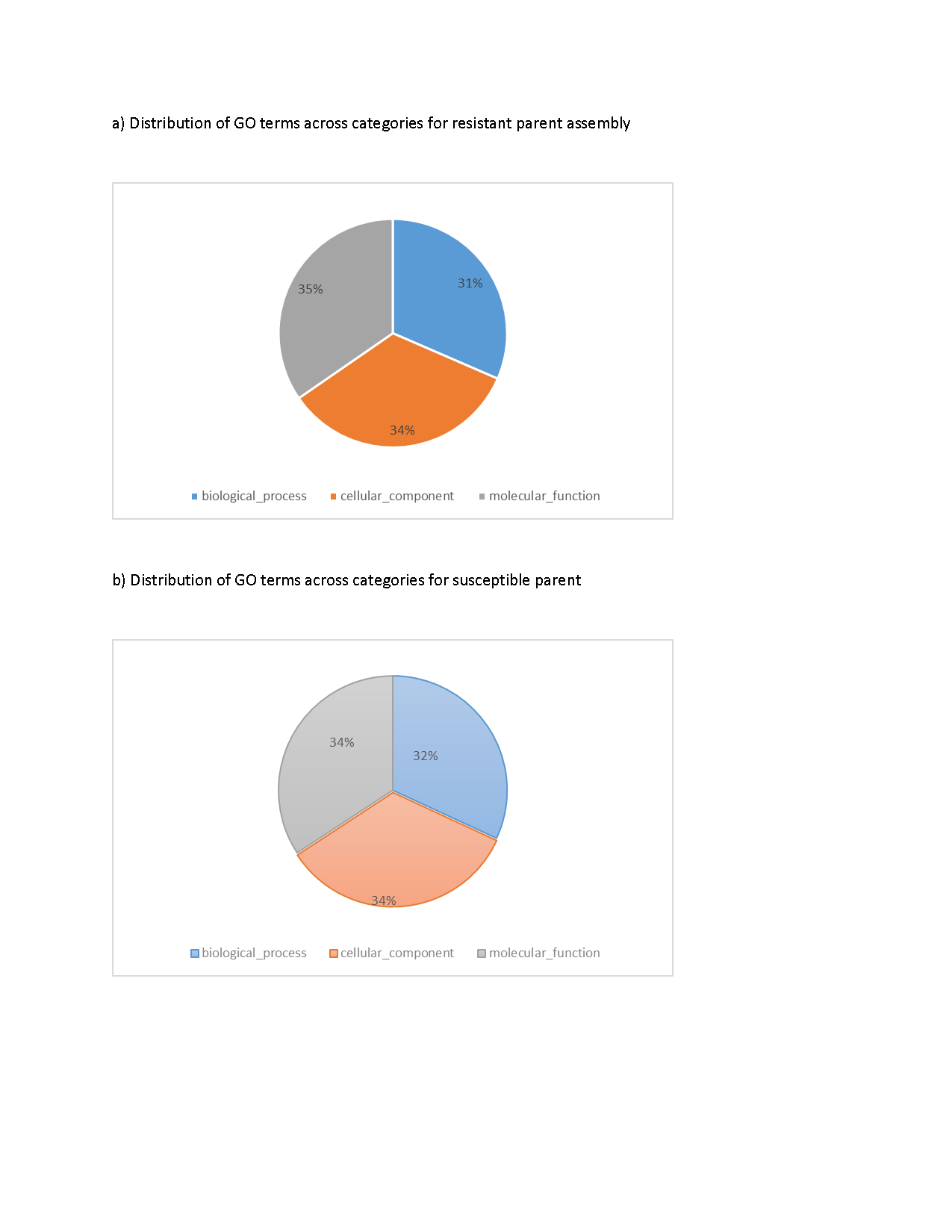

Supplement: S1 Fig — (TIF) [file pone.0215492.s001.tif]
